# Changed Profile of Serum Transferrin Isoforms in Primary Biliary Cholangitis

**DOI:** 10.3390/jcm9092894

**Published:** 2020-09-08

**Authors:** Agnieszka Grytczuk, Alicja Bauer, Ewa Gruszewska, Bogdan Cylwik, Lech Chrostek

**Affiliations:** 1 Department of Laboratory Diagnostics, University Clinical Hospital, 15-540 Bialystok, Poland; agnieszka.grytczuk@onet.eu; 2 Department of Biochemistry and Molecular Biology, Centre of Postgraduate Medical Education, 01-813 Warsaw, Poland; alabauer@wp.pl; 3 Department of Biochemical Diagnostics, Medical University of Bialystok, 15-269 Bialystok, Poland; gr_ewa@interia.pl; 4 Department of Pediatric Laboratory Diagnostics, Medical University of Bialystok, 15-274 Bialystok, Poland; cylwikb@umb.edu.pl

**Keywords:** capillary electrophoresis, glycosylation, primary biliary cholangitis, transferrin isoforms

## Abstract

Liver damage affects the synthesis of proteins and glycoproteins, and alters their posttranslational modification, such as glycosylation changing the serum profile of glycoprotein isoforms. The retention of hydrophobic bile acids in the course of cholestatic liver diseases is a major cause of liver damage in primary biliary cholangitis (PBC). The study objective was to determine the serum profile of transferrin isoforms in primary biliary cholangitis and compare it to transferrin isoforms profile in extrahepatic cholestasis. The study was carried out in 76 patients with PBC and 40 healthy blood donors. Transferrin isoforms were analyzed by the capillary electrophoresis method. The mean relative concentrations of disialotransferrin and trisialotransferrin in PBC were significantly lower than those in the healthy subjects (*p* < 0.001, *p* = 0.011; respectively). None of the transferrin isoforms changed according to the disease severity evaluated by the Ludwig scoring system. However, the disease stage affected the activity of alkaline phosphatase (ALP) and γ-glutamyl transferase (GGT), and albumin level (*p* = 0.002; *p* = 0.013 and *p* = 0.005, respectively). Our results indicate that serum profile of transferrin isoforms alters primary biliary cholangitis and differs in comparison to transferrin isoforms profile in extrahepatic cholestasis. The decreased concentrations of lower sialylated isoforms of transferrin (low percentage share in total transferrin level) are not associated with the histological stage of disease.

## 1. Introduction

Primary biliary cholangitis (PBC) is a chronic liver disease characterized by damage of small intrahepatic bile ducts resulting in cholestasis, inflammation, and bile acids (BAs) retention [1]. The retention and accumulation of hydrophobic bile acids, such as cholic acid and chenodeoxycholic acid inside cells, can damage hepatocyte in different ways [2]. Prolonged cholestasis may lead to cirrhosis and portal hypertension. BAs may disrupt cell membranes and can promote the generation of reactive oxygen species (ROS) in human liver [3]. ROS generation induces oxidative cell damage causing mitochondrial dysfunction and endoplasmic reticulum stress in isolated rat hepatocytes [4], isolated rat liver mitochondria [5], and isolated human mitochondria [6]. The rough endoplasmic reticulum (RER) is the place of biosynthesis of glycoprotein’s oligosaccharides [7]. Therefore, bile-acid-induced damage in plasma membranes and cell organelle membranes may alter the synthesis of oligosaccharides and subsequently glycoproteins in hepatocytes. Hepatocyte membrane injury induced by BAs can be structural and also functional. The structural alterations involve the aggregation between BAs and lipids in the outer membrane monolayer and consequently the formation of transient holes, which disrupt membrane integrity and resolve of hepatocytes [8]. A key tool for diagnosis PBC and differentiation between PBC, PSC (Primary sclerosing cholangitis) and AIH (Autoimmune hepatitis) is liver biopsy [9,10].

The aim of the current study was to provide evidence that primary biliary cholangitis disrupts the function of hepatocytes, resulting in alterations of protein synthesis and glycosylation in the example of transferrin. Transferrin is a negative acute-phase protein, whose concentration decreases during inflammatory disorders [11]. Discrepancy between the plasma concentrations of different acute-phase proteins is common. These variations indicate that the components of the acute-phase response are individually regulated. The changes in transferrin concentration reflect mainly the synthesis in hepatocytes. The source of microheterogeneity is its structure because transferrin is composed of amino acids and carbohydrates. Transferrin exists as a number of different isoforms resulting from variations in glycosylation, especially sialylation of oligosaccharide chains [12]. Depending on the number of sialic acid residues attached to the oligosaccharide chain, nine isoforms of transferrin can be identified, from asialotransferrin to octasialotransferrin. The synthesis and glycosylation of acute-phase proteins including transferrin is regulated at the transcriptional and post-transcriptional level [13]. Post-translational changes in the glycosylation of plasma proteins during diseases include alterations in oligosaccharide branching [14], changing sialylation [15], and decreased galactosylation of IgG (Immunoglobulin G) [16]. The changes in glycosylation develop independently of changes in the synthesis of acute-phase proteins.

We expect that the changes in the serum profile of transferrin isoforms are likely to be disease-specific and can therefore additionally confirm the toxic effect of bile acid retention on the organelle membranes of hepatocytes in primary biliary cholangitis.

## 2. Patients and Methods

### 2.1. Patients

Serum samples were collected from 76 patients with primary biliary cholangitis (PBC) (68 women, 8 men; median age for women: 53 years, range 22–75 years: median age for men: 46 years, range 34–57 years) who were hospitalized at the Department of Gastroenterology, Hepatology and Clinical Oncology of the Centre of Postgraduate Medical Education (Warsaw, Poland). Additionally, serum samples from 44 patients with carcinoma of Vater’s ampulla as an example of extrahepatic cholestasis were analyzed (females: 19, males: 25; mean age: 63.2 years; range: 43–84). The patients were hospitalized in the II Department of General and Gastroenterological Surgery, Medical University of Bialystok. The samples were frozen at −86 °C and tested immediately after thawing. Serum samples were also collected from 40 healthy adult blood donors at the Warsaw Blood Bank. All subjects gave their informed consent for inclusion before they participated in the study. The study was conducted in accordance with the Declaration of Helsinki, and the protocol was approved by the Ethics Committee of Medical University in Bialystok (Protocol number R-I-002/563/2019). The laboratory characteristics of the PBC patients and healthy controls are presented in Table 1.

The prevalence of autoantibodies in the tested patients was as follows: anti-Sp100—35.53% (27/76), anti-gp210 and anti-Ro52—34.21%, anti-centromere—7.89%, anti-p61—5.26% and AMAM2—84.21%. PBC was diagnosed using the European Association for the Study of the Liver (EASL) guidelines [1]. Because it was the first diagnosis for these patients, they had not been treated before. The differentiation with PSC was made on the basis of histopathological study of liver biopsy. The persons with suspicion of AIH or PBC-AIH were pre-eliminated. The histological scoring system was performed according to Ludwig’s staging system. The numbers of PBC patients in stages of Ludwig’s scale were: 13 in stage 1, 36 in stage 2, 16 in stage 3, and 11 in stage 4. Patients with abnormal liver biochemistry were examined by ultrasound and serological screening for AMA (Antimitochondrial antibody) and PBC-specific-ANA (presence of nuclear dots or perinuclear rims) was performed with immunofluorescence detection. Biochemical tests of all PBC patients suggested cholestatic liver disease. All patients had a liver biopsy. The main criteria included biochemical evidence of cholestasis with elevation of alkaline phosphatase (ALP) activity, presence of AMA and/or anti 2-oxoacid dehydrogenase complex and/or PBC-specific-ANA, histopathologic evidence of characteristic cholangitis, and destruction of small or medium-sized bile ducts.

### 2.2. Autoantibodies Assay

All autoantibodies except for anti-p62 were detected using commercially available ELISA kits according to manufacturer’s instructions: anti-Sp100 antibodies using IMTEC-Sp100-Antibodies kit (ITC 660040; IMTEC, Berlin, Germany), which uses recombinant Sp100 protein, anti-gp210 with QUANTA Lite gp210 kit (Inova Diagnostics, San Diego, CA, USA), which uses a highly purified peptide corresponding to an immunodominant portion of the gp210 protein, anti-AMA M2 using IMTEC-AMA M2 kit (HUMAN-IMTEC, Berlin, Germany), anti-Ro-52 with QANTA Lite SS-A 52 ELISA kit (Inova Diagnostics, San Diego, CA, USA), and anti-centromere using ACA—QANTA Lite Centromere ELISA kit (CENP-A & CENP-B) (Inova Diagnostics, San Diego, CA, USA). Optical density (OD) was measured with an automatic plate reader (Multiscan RC, Labsystem, Vantaa, Finland). Detection of anti-p62 antibodies was performed according to the procedure described by Bauer and Habior [17].

### 2.3. Transferrin Isoforms Assay

Transferrin isoforms were determined by the capillary electrophoresis (CE) assay on a MINICAP (Carbohydrate deficient transferrin) electrophoretic system using the MINICAP CDT reagent kit (Sebia, Evry, France). The transferrin isoforms are separated by their electrophoretic mobility in an alkaline buffer with a specific pH 8.8. In this method, the following major fractions can be detected: asialotransferrin, disialotransferrin, trisialotransferrin, tetrasialotransferrin, and pentasialotransferrin.

### 2.4. Total Transferrin Assay

Total serum transferrin concentration was determined by the immunoturbidimetric method using the transferrin kit from Abbott (Abbott Laboratories, Abbott Park, IL, USA) on the Architect ci8200 analyzer (Abbott Laboratories, USA). In this method, specific antibodies to human transferrin form insoluble complexes and the result as a turbidity is measured on the analyzer.

### 2.5. Total Bile Acid Assay

Total bile acids were determined by enzyme cycling method using Diazyme Total Bile Acids Assay kit (Diazyme Laboratories, Gregg Court, Poway, CA, USA). Enzyme 3-α-hydroxysteroid dehydrogenase (3- α-HSD) converts bile acids to 3-keto steroids and thio-NADH (Nicotinamide adenine dinucleotide hydride, in the presence of thio-NAD (Nicotinamide adenine dinucleotide)). The reaction is reversible. In the presence of excess NADH, the enzyme cycling occurs efficiently and the rate of formation of thio-NADH is determined by measuring specific change of absorbance at 405 nm on Indiko Plus analyzer (Thermo Fisher Scientific, Thermo, Finland).

### 2.6. Statistics

The results were expressed as mean and SD. The differences between the tested group and control were evaluated using the Mann–Whitney U test. The effect of the histological changes on the concentration of isoforms was tested by the ANOVA rank Kruskal–Wallis test. The Spearman’s rank correlation coefficient was used to assess the correlation between transferrin isoforms and laboratory tests. The differences were considered statistically significant at *p* < 0.05.

## 3. Results

**Figure 1:**
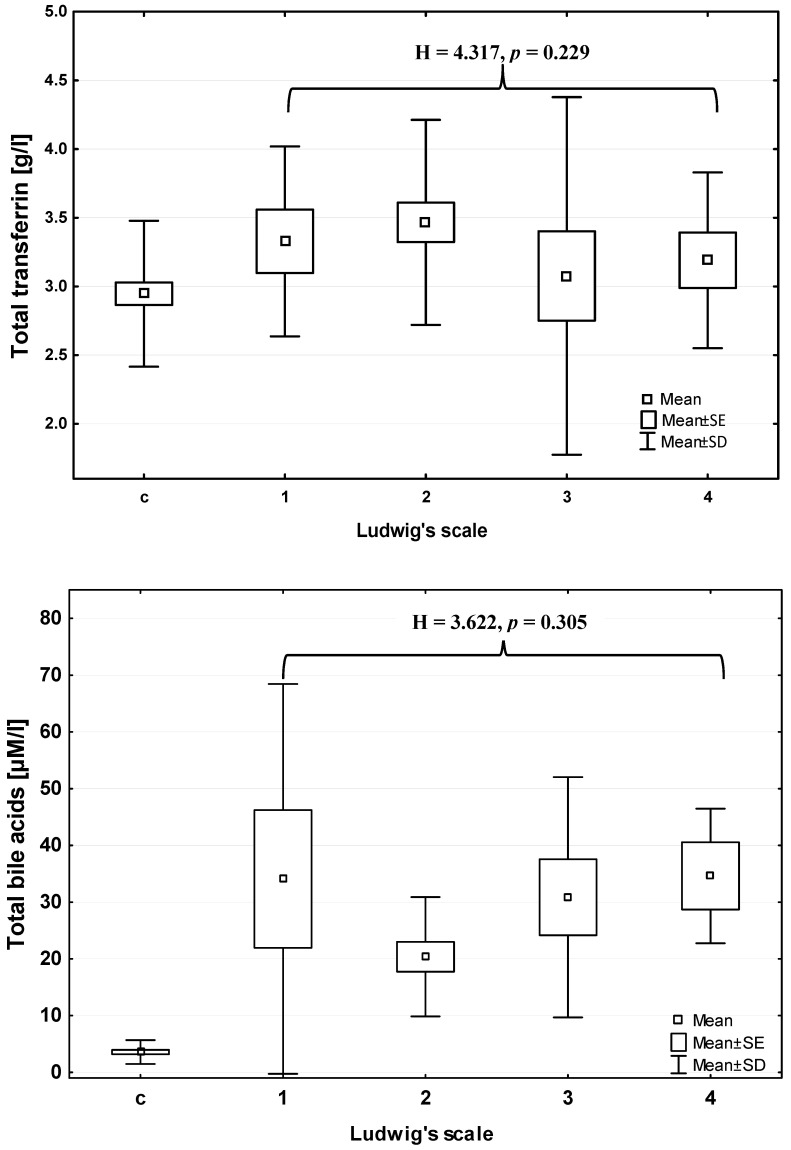
Total transferrin concentration (**top**) and bile acids (**bottom**) in PBC patients according to Ludwig’s scale. C—control group, 1–4—stages of liver injury according to Ludwig’s scale. Comparison between stages of Ludwig’s scoring system: ANOVA rank Kruskal–Wallis test (H, *p*).

Total bile acids concentration (BAs) increased significantly in PBC patients in comparison with control group (Mann–Whitney U test: Z = 6.950; *p* < 0.001) (Table 1), however did not change with the advancement of liver histological failure (ANOVA rank Kruskal–Wallis test: 3.622; *p* = 0.305) (Figure 1). The mean serum BAs concentration in extrahepatic cholestasis (carcinoma of Vater’s ampulla) was significantly higher than that in the control group (about 30 times higher) and in the PBC group (about three times higher) (Mann–Whitney U test: *p* < 0.001 for both comparisons) (Table 1). Furthermore, the total transferrin concentration (TRF) significantly increased in patients in comparison to the control group (Mann–Whitney U test: Z = 2.353; *p* = 0.019) (Table 2), and also did not change according to histopathological scale of liver injury (ANOVA rank Kruskal–Wallis test: H = 4.317; *p* = 0.229) (Figure 1). The mean serum TRF concentration in extrahepatic cholestasis was significantly lower than that in the healthy controls and PBC patients (*p* < 0.001 for both comparisons; Mann–Whitney U test) (Table 2). 

**Figure 2:**
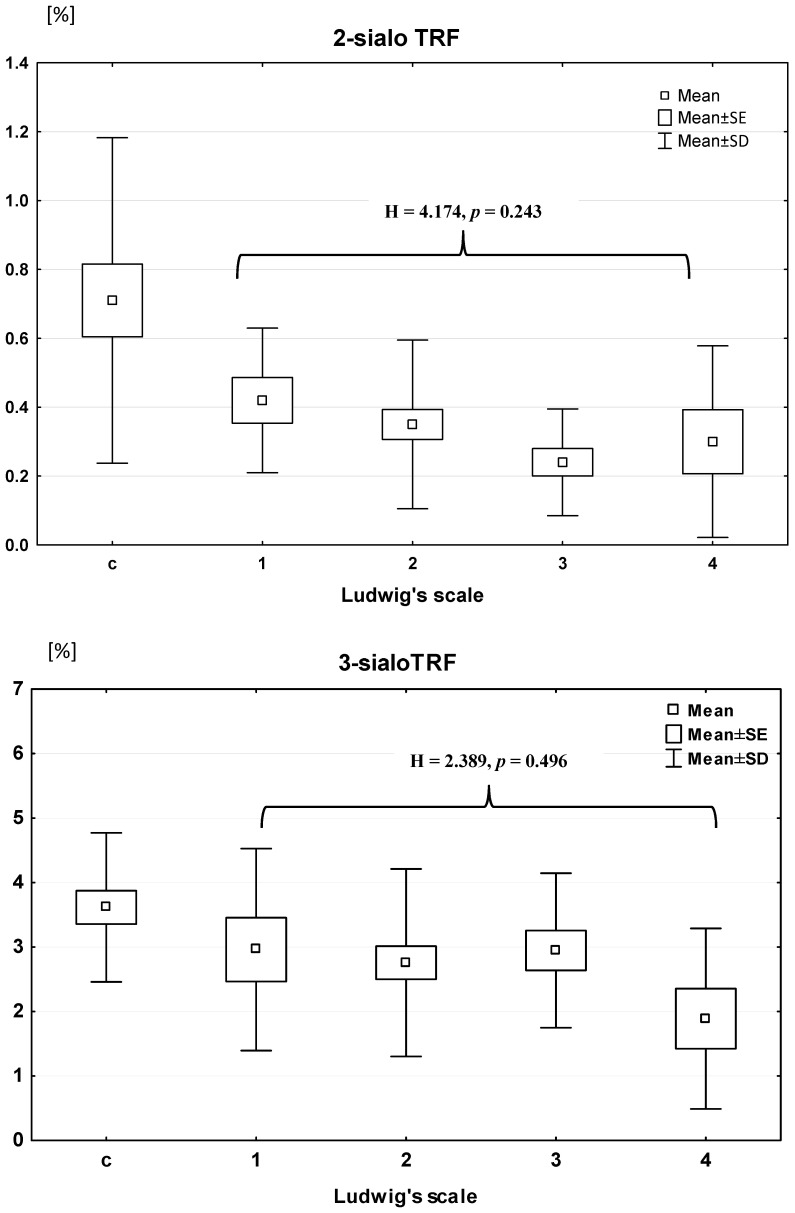
The relative concentration of 2-sialotransferrin (**top**) and 3-sialotransferrin (**bottom**) in PBC patients according to Ludwig’s scale. C—control group, 1–4—stages of liver injury according to Ludwig’s scale, SE—standard error. Comparison between stages of Ludwig’s scale: ANOVA rank Kruskal–Wallis test (H, *p*).

There were significant differences in the mean concentrations of disialotransferrin and trisialotransferrin in patients with PBC in comparison with the control group (Mann–Whitney U test: Z = −4.356; *p* < 0.000 and Z = −2.522; *p* = 0.011; respectively) (Figure 2, Table 2). The mean disialotransferrin and tetrasialotransferrin concentrations in extrahepatic cholestasis were significantly higher than that in PBC group (Z = 6.289; *p* < 0.001 and Z = 4.941; *p* < 0.001, respectively) and the concentration of pentasialotransferrin was significantly lower than that in PBC patients (Z = −5.936; *p* < 0.001) (Table 2). The mean relative concentrations of disialotransferrin and trisialotransferrin were significantly lower in PBC patients (mean: 0.33 ± 0.22% and 2.71 ± 1.42%, respectively) than those in the controls (mean: 0.935 ± 1.15% and 3.61 ± 1.16%, respectively). The relative concentrations of other transferrin isoforms (tetra- and pentasialotransferrin) did not differ in comparison to the control group.

None of the transferrin isoforms changed according to histological staging based on the Ludwig classification (ANOVA rank Kruskal–Wallis test: H = 4.17 and *p* = 0.24 for disialotransferrin; H = 2.39 and *p* = 0.49 for trisialotransferrin; H = 5.50 and *p* = 0.14 for tetrasialotransferrin; H = 6.10 and *p* = 0.11 for pentasialotransferrin). Among other tests, the histological progression of PBC exerts an impact on ALP and GGT activity (H = 14.73 and *p* = 0.002; H = 10.83 and *p* = 0.013, respectively) and on albumin concentration (H = 12.96 and *p* = 0.005). Post-hoc analysis revealed that the activity of GGT and ALP in stage 3 was higher than that in stage 1 (*p* = 0.008 and *p* = 0.006, respectively) but albumin level in stage 3 was lower than in stage 2 (*p* = 0.039). Furthermore, ALP activity in stage 4 was also significantly higher than in stage 1 (*p*= 0.015).

There was no correlation of bile acids concentration with total transferrin and individual fractions of transferrin (*p* > 0.05 for all comparisons in Spearman’s rank correlation coefficient test).

## 4. Discussion

It is generally accepted that primary biliary cholangitis (PBC) is an autoimmune liver disease characterized by the presence of specific autoantibodies. A review of several studies showed the prevalence of anti-mitochondrial antibodies (AMAs) in the course of PBC at the level of 90–95% [18,19]. In our study, AMAs were present in 84.2% of PBC patients, being a little lower than the general AMA range in PBC patients, though comparable with the frequency of autoantibodies in other studies [20]. The frequency of anti-nuclear antibodies (ANA) including anti-gp210, anti-SP100, and anti-Ro52 reached a value between 34.2% and 35.5%.

In addition to ALP, other liver biochemical tests are used to diagnose PBC, including alanine (ALT) and aspartate aminotransferase (AST), immunoglobulins (mainly IgM), and bilirubin [21,22]. Since we know that bile acid retention in cholestatic liver diseases may disrupt cell and organelle membranes and that the synthesis of glycoprotein oligosaccharides takes place in the RER membrane, we made an attempt to test the glycosylation profile of serum transferrin in the course of PBC.

Several pathways are involved in bile-acid-induced hepatocyte injury, e.g., promotion of reactive oxygen species generation [6], induction of hepatocyte necrosis and apoptosis [23], and disruption of plasma membrane integrity by BA-lipids aggregates [8]. Whatever it is, the mechanisms involved in BAs toxicity are not fully understood. Regardless of cellular and molecular mechanism of hepatocyte injury caused by the retention of hydrophobic bile acids, hepatocyte swelling and the intracellular accumulation of bile pigments have been observed [24], resulting in bile-acid-mediated inflammation in the liver. Because of this, we can expect the decrease in blood level of negative acute-phase proteins e.g., transferrin. Unexpectedly, we observed a statistically significant increase of transferrin concentration in the sera of patients with PBC. This may mean that it is more effective at damaging the cell membrane than organelle membranes, which reduces the synthesis of glycoproteins. Finally, the efflux of transferrin through membrane holes comprised of BA-lipids aggregates is more effective than impaired synthesis of transferrin in ER and Golgi apparatus.

The results of the serum profile of transferrin isoforms obtained in PBC patients are surprising. Except for changes in lower sialylated isoforms of transferrin (disialo and trisialotransferrin), we found no alterations in higher sialylated isoforms (tetra- and pentasialotransferrin). The most visible change was observed in the relative concentration of disialotransferrin showing almost a three-fold reduction in PBC patients in comparison to the controls. Despite this large change in the relative concentration of disialotransferrin, there were no shifts related to histological staging evaluated by the Ludwig scoring system. The decrease in relative concentration of trisialotransferrin was much smaller (about 25% lower). Unexpectedly, we noticed an impact of the histological progression of disease on ALP and GGT activity and on albumin concentration, perhaps because ALP and GGT are membrane-bound extracellular enzymes and are more dependent on bile duct obstruction, whereas albumin synthesis takes place in different hepatocyte compartments starting in ribosomes, continuing in endoplasmic reticulum, and ending in Golgi apparatus. It should be emphasized that only those isoforms of transferrin that constitute a very small fraction of the total transferrin concentration were reduced. The mean relative concentrations of disialotransferrin and trisialotransferrin in healthy people get a value of 0.935 ± 1.15% and 3.61 ± 1.16% (in our study), respectively. Therefore, the changes in these low-sialylated isoforms of transferrin did not affect the total transferrin concentration in PBC patients. Because of the low concentration of low-sialylated transferrin isoforms, neither of them correlated with total transferrin concentration. Furthermore, none of these isoforms correlated with total concentration of bile acids. 

The reduction in the concentration of low-sialylated isoforms in PBC patients was not accompanied by the changes in higher sialylated isoforms. The relative concentration of tetra- and pentasialotransferrin did not change. In our previous studies, we showed that the shift in one isoform of transferrin causes the reverse shift in the other, e.g., the increased level of tetrasialotransferrin was accompanied by the decreased level of pentasialotransferrin in the course of chronic hepatitis [25], and in pancreatic cancer [26], or mixed effect in the form of a significant decrease in tri- and pentasialotransferrin and a significant increase in tetrasialotransferrin in patients with rheumatoid arthritis [27]. Opposite changes in transferrin isoforms aim to maintain a stable level of transferrin. However, in liver cirrhosis (alcoholic and nonalcoholic origin) and in toxic hepatitis the changes were limited to one transferrin isoform [28]. Increased levels of trisialotransferrin in cirrhosis and disialotransferrin in toxic hepatitis were observed. Taking into consideration that in all steps of synthesis and glycosylation of proteins enzymes are involved, it means that disease-specific changes in enzyme activity are responsible for the specific profile of transferrin isoforms in the blood during the course of disease. Thus, the comparison of the serum profile of transferrin isoforms in different diseases can provide information about specific diseases, as this profile is unique to the specific disease.

The profile of transferrin isoforms in PBC patients was compared with the transferrin profile in extrahepatic cholestasis. We observed the different changes in sialylation of transferrin in these patients to those in PBC patients. Firstly, the level of disialotransferrin was not changed. Secondly, we noticed the significant changes in the concentration of higher sialylated isoforms. Thus, the tetrasialotransferrin level was significantly higher than that in the healthy controls and was higher than that in PBC patients, but the level of pentasialotransferrin was significantly lower than that in the control group and in the PBC group.

## 5. Conclusions

We can state that the serum profile of transferrin isoforms in primary biliary cholangitis is specific and differs in comparison to extrahepatic cholestasis. The decreased concentrations of lower sialylated isoforms of transferrin (low percentage share of total transferrin level) are not associated with the parallel changes in the concentrations of higher sialylated isoforms (high percentage share of total transferrin level) and are not parallel to the histological stage of disease.

## Figures and Tables

**Table 1:** Characteristics of healthy, cholestasis, and primary biliary cholangitis (PBC) groups.

	Healthy Blood Donors (*n* = 40)	Extrahepatic Cholestasis (*n* = 44)	PBC (*n* = 76)
Age (range)	35 (20–60)	63 (43–84)	52 (22–75)
Sex (F/M)	30/10	19/25	68/8
AST (µkat/L)	0.38 ± 0.40	1.85 ± 2.03 *	2.2 ± 1.2 *#
ALT (µkat/L)	0.33 ± 0.50	1.28 ± 2.12 *	2.3 ± 2.1 *#
ALP (µkat/L)	0.80 ± 0.40	11.9 ± 10.2 *	4.5 ± 3.8 *#
GGT (µkat/L)	0.40 ± 1.0	7.34 ± 8.89 *	7.8 ± 6.0 *#
Bilirubin (µmol/L)	17.1 ± 5.4	91.5 ± 123.3 *	21.5 ± 35.5 *#
Albumin (g/L)	42 ± 30	32.6 ± 6.3 *	26.7 ± 16.0 *#
BAs (µM/L)	3.57 ± 2.11	91.6 ± 37.9 *	27.97 ± 21.5 *#
AMA-M2 (+/−) (%)	ND	ND	64/76 (84.2)
Anti-gp210 (+/−) (%)	ND	ND	26/76 (34.2)
Anti-SP100 (+/−) (%)	ND	ND	27/76 (35.5)
Anti-Ro52 (+/−) (%)	ND	ND	26/76 (34.2)
Anti-p62 (+/−) (%)	ND	ND	4/76 (5.2)
Anti-centromere (+/−) (%)	ND	ND	6/76 (7.9)

Data are means ± SD (Standard deviation). * Statistically significant difference in comparison to the control group. # Statistically significant difference between the groups (cholestasis vs. PBC). ND, not detectable; AST, aspartate aminotransferase; ALT, alanine aminotransferase; ALP, alkaline phosphatase; GGT, γ-glutamyl transferase; BAs, bile acids.

**Table 2:** Transferrin and its isoforms in healthy blood donors, cholestasis, and PBC group.

	Healthy Blood Donors (*n* = 40)	Extrahepatic Cholestasis (*n* = 44)	PBC (*n* = 76)
TRF (g/L)	2.94 ± 0.53	2.26 ± 0.67 *	3.26 ± 0.86 *#
2-sialoTRF (%)	0.71 ± 0.47	0.68 ± 0.38	0.33 ± 0.22 *#
3-sialoTRF (%)	3.61 ± 1.16	3.08 ± 1.38 *	2.70 ± 1.43 *
4-sialoTRF (%)	76.84 ± 5.62	81.11 ± 2.34 *	74.92 ± 13.78 #
5-sialoTRF (%)	18.61 ± 6.03	15.15 ± 2.63 *	22.03 ± 14.25 #

Data are means ± SD. * Statistically significant difference in comparison to the control group. # Statistically significant difference between groups (cholestasis vs. PBC). TRF, total transferrin concentration.
